# Analysis of Analgesics and Adjuvant Medications on Psychiatric Comorbidities in Patients With Complex Regional Pain Syndrome 1

**DOI:** 10.7759/cureus.75629

**Published:** 2024-12-13

**Authors:** Ashley Deng, Eduardo D Espiridion

**Affiliations:** 1 Psychiatry, Drexel University College of Medicine, West Reading, USA; 2 Psychiatry, Reading Hospital Tower Health, West Reading, USA

**Keywords:** anticonvulsants, antidepressant, chronic pain, chronic regional pain syndrome, large retrospective cohort, opioid analgesics

## Abstract

Complex regional pain syndrome (CRPS) is a chronic pain disorder characterized by severe, disproportionate pain relative to an inciting event. The disorder’s pathophysiology is complex, involving both central and peripheral nervous system alterations, alongside genetic, inflammatory, and psychological factors. Using data from TriNetX, this study investigated the impact of analgesic and adjuvant therapies on psychiatric outcomes in CRPS patients. The analysis included 1,029 patients treated with non-opioid versus opioid analgesics and those using antidepressants versus anticonvulsants. Results revealed no significant differences in major depressive disorder (MDD), anxiety, suicidal ideation, or post-traumatic stress disorder (PTSD) between opioid and non-opioid analgesic groups. However, opioid analgesic was associated with a lower risk of behavioral disorders due to psychoactive substance use disorder (0.732; 95% confidence interval [CI] 0.555-0.899). Anticonvulsants, compared to antidepressants, were linked to significantly higher odds and hazards of psychiatric comorbidities: depression (odds ratio [OR] 5.475), anxiety (OR 1.87), PTSD (OR 1.551), and suicidal ideation (OR 2.718). Hazard ratios also showed higher risks for antidepressants in depression, anxiety, and PTSD. These findings highlight the risks and benefits associated with opioid analgesics and the potential therapeutic effects of antidepressants in patients with CRPS. Treatment should consider physiological and psychiatric symptoms, as both are impactful on pain management.

## Introduction

Complex regional pain syndrome (CRPS) is a chronic pain disorder that is characterized by disproportionate levels of spontaneous and evoked regional pain beyond what would be expected given the trauma. CRPS can be divided into CRPS 1 or CRPS 2 [1]. Though there is similar symptomatology, the two subtypes are divided based on causes. CRPS 1 is due to an unknown major peripheral nerve injury and accounts for 90% of cases. CRPS 2 is associated with peripheral nerve injury, which was elucidated on a nerve conduction test. An abnormal nerve conduction test is not specific to CRPS 2 but can be part of the diagnostic criteria [2]. This study will focus on CRPS 1 due to the prevalence compared to CRPS 2. Diagnosis of CRPS is based mainly on the *Budapest Criteria*, which includes *continuing pain, which is disproportionate to any inciting event*, and at least one symptom in the categories of sensory (hyperesthesia or allodynia), vasomotor (temperature asymmetry or skin color asymmetry), sudomotor/edema (edema or sweating changes), and motor/trophic (decreased range of motion, motor dysfunction, or trophic changes) [3]. A large population study of 600,000 in the Netherlands found that females aged 60-70 were most commonly affected and the upper extremity was more frequently affected than the lower [4]. Additionally, in another population-based study in the United States, fracture was cited as the most common antecedent in 46% of CRPS patients [5].

The pathophysiology of CRPS 1 is complicated and multifactorial, affecting both the central and peripheral nervous system [6]. Other factors like genetic predisposition, inflammation, and psychology, including stress, anxiety, depression, and past trauma, can influence both the severity and progression of CRPS 1, resulting in diverse presentations that vary from individual to individual. After injury, the nervous system may become hypersensitive to pain (central sensitization) and peripheral nociceptors may exhibit increased responsiveness (peripheral sensitization) [7]. These changes are mediated by inflammatory mediators and neuropeptides like substance P and glutamate, contributing to chronic pain, hyperalgesia, and allodynia. The pain inhibitory pathways of the brain which are opioid-mediated can be impacted in CRPS 1 patients, causing greater severity of pain. Disinhibition of the primary motor cortex and reduced gray matter volume in pain-related parts of the brain (insula and cingulate cortex) have also been noted [8]. Oxidative stress markers have also been shown to elevate following limb reperfusion [9]. Excessive B cell activation, along with increased IL-1β, substance P, and bradykinin, among other mediators, can influence inflammation and the immune response [1].

Psychiatric comorbidities can also play a crucial role in understanding CRPS 1. It is well-documented that chronic pain and psychiatric comorbidities exist in a bidirectional state where pain exacerbates psychiatric symptoms and vice versa [10]. The bidirectional model underscores the importance of addressing both the physical and psychiatric components of CRPS 1 in treatment. Major depressive disorder, extreme pain severity, and suicidal ideation were especially prevalent in the CRPS patient population [11]. Post-traumatic stress disorder (PTSD) is also more frequent in patients with CRPS 1 than it is in the general population [12]. Treatment of CRPS 1 is difficult as there is not one known cause. There are also limited randomized controlled trials that study treatment for CRPS 1 specifically. A meta-analysis of pharmacologic treatment options for CRPS 1 cites anticonvulsants, bisphosphonates, corticosteroids, muscle relaxants, nonsteroidal anti-inflammatory drugs (NSAIDs), opioids, and antidepressants. However, few of these treatments were studied in CPRS patients. Most are based on other neuropathic pain trials. Even fewer are randomized controlled trials [13]. We seek to add to a limited body of literature surrounding the pharmacological treatment of CRPS 1 patients, specifically studying their impact on the psychiatric comorbidities most commonly associated with CRPS 1.

## Materials and methods

Data set

This study utilized data from TriNetX, a global network of electronic health records covering over 250 million records. The de-identified data included information on lab results, diagnoses, medications, procedures, and patient demographics. Institutional Review Board (IRB) approval was not required for this study. The platform was accessible through participating institutions. Data queries were conducted using the International Classification of Diseases, Tenth Revision (ICD-10), and Veterans Affairs Drug classifications.

Study population 

The research focused on the United States Collaborative Network within the TriNetX database, involving 69 healthcare organizations. We included diagnoses from the last 10 years, specifically from January 1, 2014. The initial group included patients diagnosed with CRPS 1 (ICD-10 code G90.5) who were treated with either opioid analgesics (CN101) or non-opioid analgesics (CN103). To investigate the use of adjuvant medications, the study population was further refined to include patients with CRPS who were prescribed either anticonvulsants (CN400) or antidepressants (CN600).

Study design 

The study consisted of two main arms: one for analgesics and another for adjuvant medications. In the analgesic arm, Cohort 1, the reference group, included CRPS 1 patients who exclusively received non-opioids, while Cohort 2 included those who only received opioid analgesics. In the adjuvant medication arm, Cohort 1 included CRPS 1 patients who exclusively received antidepressants, while Cohort 2 included those who only received anticonvulsants. To ensure exclusivity, the alternative drug class was excluded through the 'cannot have' function criteria in TriNetX. Additionally, through setting temporal relationships, patients could not have a diagnosis of major depressive disorder or anxiety-related disorders before their diagnosis and treatment of CRPS. Propensity score matching was employed to balance the cohorts regarding age, sex, ethnicity, and race. Chronic illnesses were not considered confounding variables due to their low prevalence within the study population, and medications were not included in the matching due to minimal differences between cohorts. The psychiatric outcomes evaluated included diagnoses of recurrent major depressive disorder (ICD-10 code F33.0); anxiety, dissociative, stress-related, somatoform, and other disorders (ICD-10 codes F40-F48); mental and behavioral disorders due to psychoactive substance use (ICD-10 codes F10-F19); PTSD (ICD-10 code F43.1); and suicidal ideations (ICD-10 code R45.851).

Statistical analysis

The TriNetX platform was used to analyze measures of association and survival. The 69 organizations included both academic and non-academic institutions across the United States but did not include all hospital systems. Odds ratios (ORs) were calculated using logistic regression to compare the cohorts. Survival analysis was conducted using the Cox proportional hazards model to determine hazard ratios (HRs). The survival probabilities at the end of the observation period were calculated and compared using the log-rank test, along with Cox hazard ratios (HRs) and proportionality tests. The log-rank test was used specifically to assess differences in time-to-event distributions between groups under the null hypothesis, utilizing chi-square analysis.

## Results

After matching for demographic characteristics, the matched cohorts were predominantly female (73%), White (70%), and non-Hispanic or Latino (60%) (Table 1).

**Table 1:** Demographic characteristics for CRPS patients separated by non-opioid analgesic and opioid analgesic (n = 1,029). Cohort 1, non-opioid analgesic; Cohort 2, opioid analgesic. A t-test was used for continuous variables, and a chi-square test was used for categorical variables. SD, standard deviation; CRPS, complex regional pain syndrome

Cohort	Variable	Mean ± SD	Patients	Cohort (%)	*P*-value	SD
Cohort 1	Age (Current age)	52.1 ± 21.7	1,029	100%	0.998	<0.001
Cohort 2	Age (Current age)	52.1 ± 17.0	1,029	100%	0.998	<0.001
Cohort 1	Age at index	47.5 ± 22.2	1,029	100%	0.965	0.002
Cohort 2	Age at index	47.6 ± 17.4	1,029	100%	0.965	0.002
Cohort 1	Female		749	72.8%	0.882	0.007
Cohort 2	Female		752	73.1%	0.882	0.007
Cohort 1	Male		244	23.7%	0.835	0.009
Cohort 2	Male		240	23.3%	0.835	0.009
Cohort 1	White		713	69.3%	0.597	0.023
Cohort 2	White		724	70.4%	0.597	0.023
Cohort 1	Black or African American		100	9.7%	0.881	0.007
Cohort 2	Black or African American		98	9.5%	0.881	0.007
Cohort 1	American Indian or Alaska Native		10	1.0%	1	<0.001
Cohort 2	American Indian or Alaska Native		10	1.0%	1	<0.001
Cohort 1	Unknown race		136	13.2%	1	<0.001
Cohort 2	Unknown race		136	13.2%	1	<0.001
Cohort 1	Native Hawaiian or Other Pacific Islander		10	1.0%	0.002	0.140
Cohort 2	Native Hawaiian or Other Pacific Islander		0	0%	0.002	0.140
Cohort 1	Unknown ethnicity		365	35.5%	0.963	0.002
Cohort 2	Unknown ethnicity		364	35.4%	0.963	0.002
Cohort 1	Non-Hispanic or Latino		613	59.6%	0.822	0.010
Cohort 2	Non-Hispanic or Latino		618	60.1%	0.822	0.010
Cohort 1	Hispanic or Latino		51	5.0%	0.679	0.018
Cohort 2	Hispanic or Latino		47	4.6%	0.679	0.018
Cohort 1	Other race		57	5.5%	0.367	0.040
Cohort 2	Other race		48	4.7%	0.367	0.040
Cohort 1	Asian		18	1.7%	1	<0.001
Cohort 2	Asian		18	1.7%	1	<0.001

There was no significant difference in OR in developing depression, psychoactive substance use disorder (SUD), anxiety, and PTSD when comparing the opioid to non-opioid analgesics because the 95% confidence interval (CI) includes 1. Interestingly, the OR for behavioral disorder due to psychoactive SUD indicates that opioid users are less likely to develop the outcome than non-opioid users, with an OR of 0.732 (95% CI 0.555-0.899) (Table 2).

**Table 2:** Odds ratios using chi-square test of psychiatric outcomes in CRPS patients: non-opioid vs. opioid analgesics. The reference group is Cohort 1, patients taking opioid analgesics. CI, confidence interval; CRPS, complex regional pain syndrome; PTSD, post-traumatic stress disorder

Condition	Odds ratio	95% CI
Major depressive disorder	1.167	0.716-1.902
Anxiety	1.201	0.937-1.539
Behavioral disorder due to psychoactive substance use disorder	0.732	0.555-0.899
PTSD	1.628	0.811-3.270
Suicidal ideation	1.814	0.833-3.949

There was no significant difference in the time to onset of MDD, anxiety, suicidal ideation, and PTSD. The 95% CI for the HRs included 1, and the *P*-values from the log-rank tests were greater than 0.05.

In contrast, the outcomes for behavioral disorders due to psychoactive SUD were significant. The HR was 0.724 (95% CI 0.542-0.968), but the log-rank test showed no significant difference in survival probabilities (χ² = 0.855, *P* = 0.355), indicating a reduced risk of behavioral disorder due to psychoactive SUD and later onset in the opioid cohort compared to the non-opioid cohort (Table 3).

**Table 3:** Hazard ratios using Cox proportional model and log-rank test of psychiatric outcomes in CRPS patients: non-opioid vs. opioid analgesics. The reference group is Cohort 1, patients with opioid analgesic χ², chi-square; CI, confidence interval; CRPS, complex regional pain syndrome; PTSD, post-traumatic stress disorder

Condition	Hazard ratio	95% CI	χ² (Log-rank)	df	*P* (Log-rank)
Major depressive disorder	1.113	0.688-1.799	0.520	1	0.471
Anxiety	1.109	0.882-1.396	3.011	1	0.083
Behavioral disorder due to psychoactive substance use disorder	0.724	0.542-0.968	0.855	1	0.355
PTSD	1.552	0.777-3.099	0.263	1	0.608
Suicidal ideation	2.142	0.931-4.926	0.230	1	0.632

After matching for demographic characteristics, the matched cohorts were predominantly female (70%), White (75%), and non-Hispanic or Latino (65%) (Table 4).

**Table 4:** Demographic characteristics of CRPS patients using either anticonvulsants or antidepressants (n = 2,567). Cohort 1, antidepressant only; Cohort 2, anticonvulsant only A t-test was used for continuous variables, and a chi-square test was used for categorical variables. SD, standard deviation; CRPS, complex regional pain syndrome

Cohort	Variable	Mean ± SD	Patients	Cohort (%)	*P*-value	SD
Cohort 1	Current age	55.0 ± 17.5	2,567	100%	0.915	0.003
Cohort 2	Current age	54.9 ± 18.3	2,567	100%	0.915	0.003
Cohort 1	Age at index	50.1 ± 17.7	2,567	100%	0.891	0.004
Cohort 2	Age at index	50.0 ± 18.5	2,567	100%	0.891	0.004
Cohort 1	Female		1,794	69.9%	0.627	0.014
Cohort 2	Female		1,778	69.3%	0.627	0.014
Cohort 1	Male		655	25.5%	0.524	0.018
Cohort 2	Male		675	26.3%	0.524	0.018
Cohort 1	White		1,912	74.5%	0.541	0.017
Cohort 2	White		1,931	75.2%	0.541	0.017
Cohort 1	Black or African American		197	7.7%	0.559	0.016
Cohort 2	Black or African American		186	7.2%	0.559	0.016
Cohort 1	American Indian or Alaska Native		10	0.4%	1	<0.001
Cohort 2	American Indian or Alaska Native		10	0.4%	1	<0.001
Cohort 1	Unknown race		317	12.3%	0.798	0.007
Cohort 2	Unknown race		311	12.1%	0.798	0.007
Cohort 1	Native Hawaiian or Other Pacific Islander		10	0.4%	1	<0.001
Cohort 2	Native Hawaiian or Other Pacific Islander		10	0.4%	1	<0.001
Cohort 1	Unknown ethnicity		804	31.3%	0.653	0.013
Cohort 2	Unknown ethnicity		819	31.9%	0.653	0.013
Cohort 1	Hispanic or Latino		93	3.6%	0.659	0.012
Cohort 2	Hispanic or Latino		99	3.9%	0.659	0.012
Cohort 1	Non-Hispanic or Latino		1,670	65.1%	0.540	0.017
Cohort 2	Non-Hispanic or Latino		1,649	64.2%	0.540	0.017
Cohort 1	Other race		99	3.9%	0.885	0.004
Cohort 2	Other race		101	3.9%	0.885	0.004
Cohort 1	Asian		32	1.2%	0.282	0.030
Cohort 2	Asian		24	0.9%	0.282	0.030

Depression was found to be more strongly linked to the anticonvulsant condition, with an OR of 5.475 (95% CI 3.459-8.665). This indicates that patients receiving anticonvulsants are much more likely to develop depression compared to those receiving antidepressants. Anxiety also showed a significant increase in risk, with an OR of 1.87 (95% CI 1.612-2.169). PTSD was similarly elevated, with an OR of 1.551 (95% CI 1.033-2.33), suggesting a moderate association. On the other hand, SUD did not show a significant increase in odds, with an OR of 1.044 (95% CI 0.896-1.216), indicating no strong link. Finally, suicidal ideation exhibited a notable increase in risk, with an OR of 2.718 (95% CI 1.313-5.627), highlighting its significant association with the anticonvulsant-only condition (Table 5).

**Table 5:** Odds ratios using chi-square of psychiatric outcomes in CRPS patients: antidepressants vs anticonvulsants. The reference group is Cohort 1, patients using antidepressants only. CI, confidence interval, CRPS, complex regional pain syndrome; PTSD, post-traumatic stress disorder

Condition	Odds ratio	95% CI
Depression	5.475	3.459-8.665
Anxiety	1.87	1.612-2.169
Behavioral disorder due to psychoactive substance use disorder	1.044	0.896-1.216
PTSD	1.551	1.033-2.33
Suicidal ideation	2.718	1.313-5.627

The HR was notably high at 5.437 (95% CI 3.447-8.576), although the log-rank test did not show statistical significance (χ² = 2.49, df = 1, and *P* = 0.1146). Anxiety exhibited a significant increase in hazard, with an HR of 1.813 (95% CI 1.583-2.077), supported by a log-rank χ² of 9.498 (df = 1, *P* = 0.0021). The risk of SUD showed no significant hazard increase, with an HR of 1.045 (95% CI: 0.908, 1.204) and a nonsignificant log-rank χ² of 0.269 (df = 1, *P* = 0.6040). PTSD was associated with a moderately elevated HR of 1.553 (95% CI 1.038-2.325), but the log-rank test did not yield significance (χ² = 0.008, df = 1, *P* = 0.9278). Finally, suicidal ideation showed a notable increase in risk with an HR of 2.72 (95% CI 1.317-5.619), although the log-rank test did not reach statistical significance (χ² = 0.258, df = 1, *P* = 0.6114) (Table 6).

**Table 6:** Hazard ratios and log-rank test of psychiatric outcomes in CRPS patients: antidepressants vs. anticonvulsants. The reference group is Cohort 1, patients using antidepressants only. χ²,  chi-square; CI, confidence interval; CRPS, complex regional pain syndrome

Condition	Hazard ratio	95% CI	χ² (Log-rank)	df	*P* (Log-rank)
Depression	5.437	3.447-8.576	2.49	1	0.1146
Anxiety	1.813	1.583-2.077	9.498	1	0.0021
Behavioral disorder due to psychoactive substance use disorder	1.045	0.908-1.204	0.269	1	0.6040
PTSD	1.553	1.038-2.325	0.008	1	0.9278
Suicidal ideation	2.72	1.317-5.619	0.258	1	0.6114

## Discussion

This study examined the association between non-opioid versus opioid analgesic and antidepressant versus anticonvulsant treatments on psychiatric outcomes in patients with CRPS 1. While the findings indicate differences in psychiatric outcomes based on the type of medication used, these results should be interpreted cautiously, emphasizing the need for further research to guide clinical decision-making in this complex patient population.

The analysis of non-opioid and opioid analgesics revealed nuanced impacts on psychiatric outcomes. While no significant differences were observed in the development of MDD, anxiety, suicidal ideation, or PTSD, patients using opioids were less likely to develop behavioral issues due to psychoactive SUD compared to those using non-opioid analgesics. This is in contrast to some studies, which have found that opioids are effective for pain management but carry a high risk of dependency and misuse, several other studies have also identified an association between prescription opioid misuse and suicidal ideation and attempts [14-16]. However, in alignment with our findings, a study encompassing 86,168 persons across the United States found that opioid use without misuse is associated with lower odds of suicidal ideation and plans [17]. Effective opioid usage in pain control could be effective in lowering suicidal ideation as well as buffering substance use disorder tendencies.

Opioid analgesics are useful in treating chronic pain [18]. A prospective study of chronic pain patients found significant pain relief over six months of opioid analgesic treatment [19]. Psychiatric associations with opioids include SUD, where patients develop a dependency that can lead to a cycle of addiction exacerbating existing psychiatric conditions [20]. Long-term opioid use can also contribute to mood disorders such as depression and anxiety, complicating the psychiatric profile of patients [21]. This is especially relevant to chronic pain patients where treatment occurs over long periods and psychiatric comorbidities can exacerbate opioid use and vice versa. Understanding risk factors for misuse is essential before the prescription of opioid analgesics. These can include untreated psychiatric disorders, age, history of abuse and addiction, and living environment [22]. Non-opioid analgesics like NSAIDs and acetaminophen are generally considered safer concerning addiction [23]. They may be just as effective as, or even more effective than, opioid analgesics in long-term treatment [24].

The study also compared psychiatric outcomes in CRPS 1 patients using antidepressants versus anticonvulsants as adjuvant therapies. Antidepressants were associated with significantly higher odds and hazards of psychiatric comorbidities, including depression, anxiety, PTSD, and suicidal ideation. SUD was not significant. This could be due to other preexisting psychiatric conditions, besides MDD or anxiety, where antidepressants are often prescribed for patients with a wide range of mood disorders, whereas anticonvulsants are conventionally used as pain management or anti-seizure [25-27]. Prospective studies are necessary to exclude all preexisting mood disorders. The marked increase in psychiatric symptoms among the antidepressant group, particularly for depression and suicidal ideation, suggests a need for careful monitoring and possibly adjunctive therapies to address these risks. 

Antidepressants serve a dual role in pain management and treating psychiatric conditions, presenting a complex relationship with psychiatric health [28]. They can sometimes exacerbate psychiatric symptoms if not taken correctly or with the appropriate dosage, as the therapeutic index for each drug is narrow [29]. Both antidepressants and anticonvulsants are typically not associated with addiction [30]. 

The findings emphasize the complexity of managing CRPS 1, highlighting the importance of tailoring the choice of analgesic to the patient's comorbid psychiatric condition for optimal outcomes. Opioids, due to their effectiveness in pain management, may have a therapeutic effect in managing substance use disorder and suicidal ideation. However, they also pose a significant risk of developing substance use disorder, highlighting the importance of cautious use and careful consideration of alternative treatments. Non-opioid analgesics, although associated with a lower risk of SUD, were not found to protect against psychiatric symptoms, suggesting that factors such as underlying pain severity and patient psychological profiles play critical roles in treatment outcomes. For adjuvant therapies, antidepressants showed a relatively safer profile regarding psychiatric outcomes; however, there is limited evidence specifically addressing the use of antidepressants as adjuvant therapy to prevent patients with CRPS 1 from developing psychiatric disorders. Both antidepressants and anticonvulsants are used to manage various psychiatric conditions and pain management, which may be relevant to CRPS 1 patients. Treatment decisions should be individualized, considering the patient’s psychiatric history and overall clinical context.

This study has several limitations, including its reliance on retrospective data, which may overlook relevant comorbidities and potentially confound the observed relationships. Furthermore, key details such as pain severity, medication dosage, adherence and modifications, treatment duration, and direct comparisons between multiple cohorts were unavailable. These limitations constrain the generalizability and robustness of the conclusions. Nevertheless, the extensive database provided an opportunity to analyze a large cohort of patients with a rare and complex condition - offering valuable insights into the association between analgesic and adjuvant treatments and psychiatric comorbidities.

Future research should prioritize prospective studies to better understand the causal relationships between treatment types and psychiatric outcomes in CRPS 1 patients. Moreover, exploring interventional treatment options such as nerve blocks, neuromodulation, and integrated pharmacological-psychological approaches could offer more comprehensive strategies for managing this complex condition.

## Conclusions

Overall, this study sheds light on a relatively under-studied disease with unique and complicated pathophysiology and treatment. Ultimately, careful consideration of the individuals’ preexisting conditions should inform the decision between opioid and non-opioid analgesic and adjuvant analgesic therapy. A holistic approach that addresses both the physiological and psychiatric aspects of CRPS 1 is essential to minimize the risk of psychiatric outcomes that can exacerbate the pain condition.
